# The Association of Acculturation and Complementary Infant and Young Child Feeding Practices Among New Chinese Immigrant Mothers in England: A Mixed Methods Study

**DOI:** 10.3390/ijerph16183282

**Published:** 2019-09-06

**Authors:** Xiaoning Zhang, Lorna Benton

**Affiliations:** 1 School of Nursing, Xuzhou Medical University, 209 Tongshan Road, Xuzhou 221004, China; 2 Great Ormond Street Institute of Child Health, University College London, 30 Guilford Street, London WC1N 1EH, UK; lorna.benton.09@ucl.ac.uk; 3 Department of Anesthesiology, Xuzhou Medical University, 209 Tongshan Road, Xuzhou 221004, China

**Keywords:** acculturation, Chinese mothers, infant and young child, complementary feeding practices, new immigrants, minority

## Abstract

Acculturation has an influence on mothers’ beliefs and the perceived behaviours of different ethnicities. Few studies have been conducted on complementary infant and young child feeding practices (CIYCFP) in minorities in England, particularly in Chinese immigrants. This mixed study aims to explore the association of acculturation and IYCF among new Chinese immigrant mothers using purposive snowball sampling from an informal Chinese community. The participants’ responses to the *Infant Feeding Style Questionnaire* (IFSQ) and *Mutual Intercultural Relations in Plural Societies* (MIRIPS), questionnaire (*n* = 32) were collected. A sub-set of 15 also participated in semi-structured interviews. Pearson’s correlation coefficient analysis and thematic analysis were performed to analyse the survey and semi-structured interview data, and triangulation was employed to integrate quantitative and qualitative findings. This study indicated that Chinese mothers who scored high in integration were more likely to respond to satiety and attention; those inclined to be marginalised were more likely to indulge their children. Those who were more culturally separated were more likely to restrict the food quality offered to their children. This study also indicated that Chinese immigrants balanced western and Chinese feeding practices to combat feeding and culture conflict. This study presents preliminary findings of the association between acculturation and CIYCFP, which can improve culturally appropriate CIYCFP in minorities. Further studies are needed to explore intervention programs to tailor CIYCFP with consideration for acculturation in the minority.

## 1. Introduction

Acculturation is “the process of cultural change that occurs when immigrants from heritage cultural backgrounds come into continuous first-hand contact with a dominant culture, [as well as the] subsequent changes in the culture of heritage patterns of either or both groups” [1]. Both culture—defined as the ideas and social behaviour of a particular group of people or society—and ethnicity are recognised determinants of health [2]. Cultural differences within England might contribute to growing ethnic health inequalities [3]. The cultural differences around health knowledge for South Asians living in high-income countries may contribute towards sub-optimal timing of complementary feeding. This, in turn, later influences differences in obesity trends [4]. The theoretical conceptualization of acculturation has changed from a simplified bipolar model to a complex, multidimensional process. This is according to how immigrants deal with the adoption or retention of (or immersion in) the heritage or dominant culture [5]. Immigrants can relate to, accommodate to and adapt to each other following contact as they carry out their daily lives in culturally diverse societies [6]. From one culture to another, negotiation and adaptation of heritage or dominant culture results in four modes: assimilation, integration, separation and marginalisation [7]. First-generation Asians experience acculturation changes in health status and dietary patterns, moving closer to those of the dominant group and the increased fat and caloric intake patterns of the United States. This leads to higher percentage of overweight or obese individuals [8,9,10].

Complementary feeding is the process that begins when breast milk is no longer enough to satisfy the nutritional needs of an infant [11]. Infants should start receiving an appropriate quality and quantity of solid foods from six months onwards. The period from 6 to 24 months of age is the time when malnutrition and obesity start in many infants [12]. Inadequate complementary infant and young child feeding practices (CIYCFP) (i.e., improper timing, quality or quantity, and appropriateness) represent major risks to the health and later development of children. These risks include long-lasting effects on cognition, school achievement and increased risks of developing non-communicable diseases, such as obesity, type II diabetes, dental caries and poor cardiovascular health. There is also a risk of the intergenerational transmission of obesity [13]. Complementary feeding not only depends on the availability of a variety of foods in the household but is also influenced by the feeding beliefs and behaviours of mothers [12]. The feeding practices of mothers can be categorised into five domains. (a) The laissez-faire mother does not limit dietary quality or quantity of the child and interacts little. (b) The pressuring/controlling mother is concerned with increasing the amount of food the child consumes and soothes child using food. (c) The restrictive/controlling mother limits unhealthy foods and the quantity of food the child consumes. (d) The responsive mother is attentive to the hunger and satiety cues of the child and monitors the dietary quality the child receives. (e) The indulgent mother does not limit the quantity or quality of food the child consumes [14]. 

Complementary feeding practices are influenced by the individual’s culture and how they uphold a child’s own eating behaviours [15]. A recent study has improved recognition of the wider range of complementary feeding practices among ethnically diverse mothers [14], while some research has indicated that acculturation and ethnicity might impact on children’s food-related beliefs, behaviours and preferences. The mother’s acculturation to the dominant culture influences breastfeeding [16,17,18,19]. Acculturation is associated with the differences of mothers’ dietary beliefs and behaviours [9]. A negative finding of acculturation is that a child’s health deteriorates the more they assimilate to the dominant culture [10]. Malnutrition in the first two years increases the risk of later developing non-communicable diseases, which cause an increasing public health burden [20]. Establishing CIYCFP beliefs and behaviours in early life is an important health strategy to combat childhood overweightness and obesity [21].

Little is known about how the acculturation of mothers may influence CIYCFP in minority communities living in England. Findings from a review emphasised the influence of acculturation on Chinese immigrant mothers’ feeding practices [22]. Chinese immigrants are the fastest-growing ethnic minority population in England [23], representing approximately 0.5% of the English population [24]. Yet, Chinese immigrants receive relatively little research attention [24] given that they exemplify the complexities of ethnicity and acculturation in minority groups for health services. Furthermore, to the best of our knowledge, no study has investigated the association between acculturation and CIYCFP among new Chinese immigrants. Without considering these cultural changes and the context of socioeconomic and environmental influences, public health strategies will remain untargeted and ineffective and lead to growing health inequalities and public expenditure. This is an important omission because current nutrition-specific interventions might be insufficient to optimise IYCF if the acculturation context in which they exist is ignored.

Considering this gap, this mixed qualitative and quantitative study explores the association between acculturation and CIYCFP among new Chinese immigrant mothers living in England. We aim to identify the barriers and facilitators to acculturation influencing CIYCFP in new Chinese immigrants in order to develop a culturally appropriate CIYCFP intervention that may be tailored to the needs of infant and young child. 

## 2. Materials and Methods 

### 2.1. Study Design

This mixed methods study used questionnaire surveys (quantitative) and semi-structured interviews (qualitative) [25] to explore the association between acculturation and CIYCFP among new Chinese immigrant mothers. We used questionnaires to measure acculturation and CIYCFP and semi-structured interviews to explore Chinese mothers’ views of acculturation and CIYCFP. Interviews are well-suited in exploring subjective attitudes and experiences because they allow for flexibility in the ordering of the questions to permit for natural rapport and sensitivity to unsolicited themes [26]. Triangulation was employed to seek corroboration between quantitative and qualitative data [27] in order to gain a more complete picture [25]. A protocol of triangulation was developed through the following steps: (a) producing a convergence coding matrix; (b) considering agreement, partial agreement, silence or dissonance [25]; and (c) listing the findings, followed by convergent, complementary or dissonant information. 

#### 2.1.1. Surveys

The basic demographic data of participants was collected at the start of the survey and included questions on age, place of birth, education level, employment status, marital status, length of stay in the UK and number of children. 

The *Infant Feeding Style Questionnaire (IFSQ)* is a validated measure [14] that can assess mother’s beliefs and behaviours regarding IYCF [28,29] and the parenting styles they adopt regarding specific feeding styles [30,31], based on concerns and constraints. The IFSQ assesses for five feeding styles: laissez-faire, pressuring/controlling, restrictive/controlling, responsive satiety, and indulgence. All five domains were coded on a 5-point Likert scale (1 = disagree, 2 = slightly disagree, 3 = neutral, 4 = slightly agree and 5 = agree). Five domains contain 13 subscales and 83 items. Of these, 39 items probed beliefs and 44 items probed behaviours (Table 1). The laissez-faire style included an assessment of attention and dietary quality, and the responsive style included an assessment of satiety and attention. Lower scores meant that mothers had higher tendencies towards the domains of laissez-faire and responsiveness. The pressuring/controlling style assessed for finishing, cereal and soothing feeding. The restrictive/controlling style assessed for the amount and quality of diet. The indulgent style assessed for permissive, coaxing, soothing and pampering feeding. Lower scores represent a weaker performance in the pressuring/controlling, restrictive/controlling and indulgence domains. Research showed that the values of internal reliability (H coefficient) were ≥0.80 for all values. The values of restrictive amounts, pressuring to finish and with cereal were ≥0.75 [14]. The internal consistency of the indulgence, pressuring, restrictive and laissez-faire diet qualities were ≥ 0.70. The responsive satiety and attention were ≥0.60 [32]. In this study, we used the H coefficient and Cronbach’s alpha to assess internal reliability (Cronbach’s alpha = 0.910). Each subitem had a coefficient of >0.898. This study validated use of the IFSQ to measure the IYCF beliefs and behaviours of Chinese mothers.

The *Mutual Intercultural Relations in Plural Societies* (MIRIPS) [7] *Questionnaire* determined how immigrants desired to acculturate into a dominant society, and it was used to measure the process of acculturation across ethnic groups by appraising a variety of acculturation positions taken by immigrants to adapt and immerse themselves within the dominant society. MIRIPS contain questions about basic information (i.e., age, sex, education, religion, socioeconomic status, ethnic origin, individual marriage preference, neighbourhood, ethnic composition and place of birth/length of residence) and 16 scales: languages known/used, social contacts, travel, cultural identity, security, acculturation attitudes and expectations, perceived discrimination, multicultural ideology, tolerance/prejudice, attitudes towards immigration, attitudes towards ethnocultural groups, psychological problems, sociocultural competence, self-esteem, life satisfaction and social desirability scale. Assimilation entails avoiding participation in the immigrant’s culture of heritage and immersing fully in the dominant society. Integration entails immersion in both the culture of heritage and dominant societies. Separation entails withdrawal from the dominant society and completely maintaining the culture of heritage. Marginalisation entails a lack of immersion either in the culture of dominant or heritage. Participants provided their responses using a five-point Likert scale (1 = disagree, 2 = slightly disagree, 3 = neutral, 4 = slightly agree and 5 = agree). Research applying MIRIPS to Chinese immigrants [33] showed that the Cronbach’s alpha values were 0.70, 0.48, 0.44 and 0.52 for integration, separation, marginalisation and assimilation, respectively [5]. This study assessed acculturation attitudes and expectations. The Cronbach’s alpha values of integration, separation, marginalisation and assimilation were 0.448, 0.467, 0.472 and 0.467, respectively. 

#### 2.1.2. Interviews

Open-ended questions (developed using a literature review) and prompts were employed to explore new Chinese immigrant mothers’ views of the association between acculturation and CIYCFP. Chinese mothers were drawn for interviews from the questionnaire survey sample, and all interviews were audio recorded. The mothers were allowed to choose their preferred language. The interviews ranged in length from 30 to 70 minutes. Data saturation was used to guide the total number of interviews conducted. 

A semi-structured interview guide was first piloted with two Chinese mothers to verify that the questions were targeted and clear, as well as encouraged and guided open thinking towards answering questions. Questions were mainly as follows. *How do you decide which foods to introduce as solid foods to your child? What kind of foods do you prefer to buy for your child? How do you decide when to stop feeding a child (portion sizes)? Have you faced any problems when feeding your child? Do you think there is a difference between Chinese and British dietary culture, and how does that influence the way you feed your children? What cultural factors affect your choices and practices in healthcare? What factors affect your choice of foods? If there is a feeding conflict between British and Chinese culture, which has a greater influence on your choice?*

### 2.2. Participant Recruitment

Chinese immigrants are typically considered a “hard to reach” minority due to geographical dispersal, language barriers and inadequate resources. It can be challenging to efficiently access enough participants for large scale quantitative studies, especially those with children aged between 6 months to 2 years. If they have 2 to 4 children, they may take care of several children at the same time, even though they are housewives. This study followed a purposive snowball sampling. We focused recruitment efforts in informal community organisations where Chinese immigrants were concentrated, such as Chinese churches in central London. This was done verbally in English and Chinese by a researcher (XNZ) and also through electronic forms on a social media application with groups for mothers (WeChat, a popular application). Interested participants contacted the researcher (XNZ) face to face, by telephone or via social media applications. A mutually convenient date, time and location were chosen with eligible participants, so they could complete the survey or interview, and these were offered in their preferred language (i.e., Mandarin or English, all materials had versions available in both languages). As compensation, all participants received gift cards worth £10 for completing the questionnaires and £5 for participating in the interview.

#### Inclusion and Exclusion

Inclusion criteria included (a) participants with self-identified Chinese origin who immigrated from mainland China; (b) who settled in England for at least one year as first-generation immigrants; (c) who have at least one child age between 6 to 24 months during the study, and (d) all carried to full term. Exclusion criteria included mothers and/or children of mixed backgrounds (i.e., White and Chinese, black and Chinese, Asian and Chinese or other combinations) and participants with any self-reported medical complications. Participant selection was undertaken by two researchers (XNZ and CB). Clarifications or disagreements over inclusion and exclusion were referred to the research team for resolution.

### 2.3. Data Collection, Management, Storage and Ethics Approved

Data collection was conducted by a researcher (XNZ) with proficiency in Mandarin and English, which maintained the validity of the cross-cultural study [34]. Potential participants were given a verbal or electronic summary of the study. If participants agreed to take part, they were provided a written participant information sheet (PIS) detailing the research aim, procedures, expected outcomes, risks and benefits and the participants’ rights not to participate. The researcher re-iterated this information throughout the identification, approach and recruitment processes. All participants were given the opportunity to provide written informed consent. If they were recruited online, we posted them all the documents and they returned the signed documents. All data were anonymised and processed as outlined by the British Educational Research Association (BERA) [35] with regards to data protection and storage of personal data. The datasets generated and analysed during the current study are not publicly available due to original consent but are available from the corresponding author on reasonable request. This study acquired ethical approval from the Institutional Review Board of University College London (Ethics Identification Number 10271001) on 15 March 2017.

### 2.4. Analysis

The descriptive variables of acculturation and CIYCFP were analysed using Stata 15 (Stata Corporation, College Station, TX) for Windows. Data was double-checked for any errors during data entry. Pearson’s correlation coefficients analysis was performed to examine the association of acculturation with CIYCFP. The missing values were not excluded and were replaced by mean values. 

All interviews were conducted and transcribed verbatim in Mandarin. During interviews, all mothers preferred Mandarin, as they thought they could better express any opinions in Mandarin than in English. Any the details of participants were de-identified. All the verbatim transcriptions were translated into English and back-translated by a bilingual researcher (XNZ) to ensure equivalence. Discrepancies between the original and the back-translated Chinese version were discussed among the bilingual translators until a consensus regarding the linguistic and cultural equivalence was reached. Initial coding, categorisation and quote storage were conducted using the NVivo 11 software package (QSR International). Thematic analysis was conducted to identify common and unique patterns that extend through the whole set of interviews, and analysed using a qualitative approach as described by Braun and Clarke [36]. We established domains from the semi-structured tool and generated codes from open fashion data (no pre-set coding). We systematically reviewed the codes in a reflexive iterative manner [37] to generate themes and sub-themes until no new findings were identified. The analysis allowed for new themes and sub-themes to emerge inductively. Data reliability was established by researchers comparing similar and congruent themes. Any differences in coding and developing themes were resolved by reviewing the interviews and further discussing them until conformity was reached. 

After the collection and analysis of the quantitative and qualitative data exclusively, we compared and contrasted results from the two phases. Findings from the survey were compared to themes and quotes from the qualitative interviews. Areas of contention and convergence between the qualitative and quantitative data were dissected in the analysis and interpretation phase. The emphasis of triangulation was placed on seeking convergence, corroboration, correspondence and dissonance of results from quantitative and qualitative study [27].

## 3. Results

### 3.1. Participant Characteristics

A total of 32 new Chinese immigrant mothers were recruited between June 2017 to March 2018. Twenty-one mothers completed the IFSQ and MIRIPS questionnaires survey on printed paper (*n* = 21) in Chinese churches. Due to convenience, 11 mothers agreed to complete the questionnaires using Google Sheets. Of the 15 Chinese mothers, majority were interviewed in private areas in Chinese churches (*n* = 9), with the remainder interviewed over WeChat voice call (*n* = 6). The mean maternal age was 33.06 (SD = 5.39) years, the number of children they had ranged from one to five. Of these 32 months, 13 (40.6%) had started or completed postgraduate studies. Two were unmarried and 22 (68.7%) were housewives. These mothers had been in England for periods ranging from 6 to 27 years.

### 3.2. Quantitative Results

#### 3.2.1. Distribution of CIYCFP and Acculturation

Table 1 and Table 2 indicate that Chinese mothers were more responsive to children’s satiety; they scored highest on responsive to satiety with a score of 3.96 (SD = 0.72). They were not very indulgent towards their children, scoring lowest on indulgence with a score of 2.15 (SD = 0.89). Chinese mothers paid more attention to response, with the highest scoring subscale being belief of responsive attention (score of 4.28, SD = 0.92). They were less soothing to indulge their children, with subscales scoring the lowest in indulgence to soothing belief (score of 1.98, SD = 0.99). Table 3 shows that scores of integration, separation, assimilation and marginalisation were 4.44 (SD = 0.62), 2.03 (SD = 0.88), 1.79 (SD = 0.57) and 1.67 (SD = 0.73), respectively.

#### 3.2.2. The Association between Acculturation and CIYCFP

**Figure 1:**
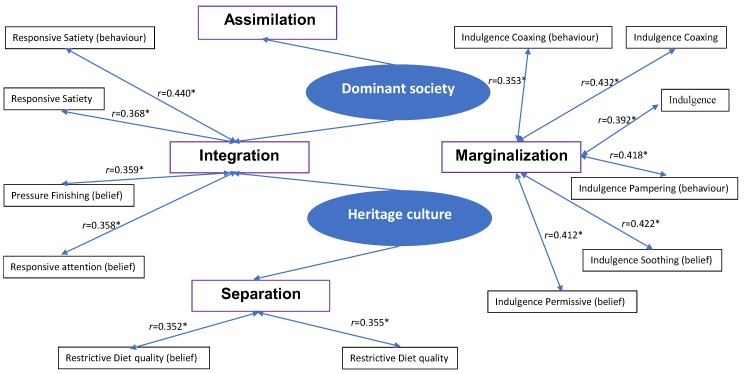
The association between acculturation and CIYCFP. * Correlation is significant at the 0.05 level (2-tailed).

Pearson Correlation coefficients analysis results of the association of acculturation and complementary infant and young child feeding practices. (Figure 1).

Table 3 indicates that the laissez-faire, pressuring, restrictive and satiety–responsiveness attitudes were not significantly correlated with integration, assimilation and separation (*r* < 0.310). Marginalisation was weakly correlated with indulgence (*r* = 0.392, *p* < 0.05). 

Table 4 demonstrates that all subscales of IYCF were not significantly correlated with assimilation (*r* < 0.170), while integration was weakly correlated with responsive satiety (*r* = 0.368, *p* < 0.05). Separation was weakly correlated with restrictive diet quality (*r* = 0.355, *p* < 0.05), and marginalisation was moderately correlated with indulgence coaxing (*r* = 0.432, *p* < 0.05). Laissez-faire attention, pressuring soothing and indulgence soothing were not significantly correlated with integration, assimilation, separation and marginalisation (*r* < 0.320).

Table 5 shows that the behaviour of laissez-faire attention, pressuring with cereal, restricting amount and diet quality and indulgence soothing were not significantly correlated with integration, assimilation, separation and marginalisation (*r* < 0.340). The behaviour of satiety responsiveness was moderately correlated with integration (*r* = 0.440, *p* < 0.05), and the behaviour of indulgence coaxing and pampering were weakly correlated with marginalisation (*r* = 0.353, 0.418; *p* < 0.05). 

Table 6 indicates that the belief of laissez-faire attention and diet quality, pressuring with cereal and soothing, satiety responsiveness and indulgence pampering were not significantly correlated with integration, assimilation, separation and marginalisation (*r* < 0.350). The belief of pressuring finishing and responsive attention were weakly correlated with integration (*r* = 0.359, 0.358; *p* < 0.05). The belief of restrictive diet quality was weakly correlated with separation (*r* = 0.352, *p* < 0.05), and the belief of indulgence permissive, coaxing and soothing were moderately correlated with marginalisation (*r* = 0.412, 0.421, 0.439 and 0.422, respectively; *p* < 0.05).

### 3.3. The Triangulation of Quantitative and Qualitative Results

#### 3.3.1. Relationship of Integration with Responsive and Pressure Feeding

Integration has widespread acceptance in a culturally diverse society. With respect to this, large numbers of interviews discussed a variety of integration related to responsive satiety, which was corroborated by the results of the quantitative analysis. For instance, mothers stated,

“My children understand that when they are full, they will not eat. If I feed them more, they would spit out and shake their heads to tell me that they do not want to eat.”

(id 15)

*“My child was picky eater. When I tried to give new food to her, I gave her the same food repeatedly. One day she would eat a bit. For example, I put cherry tomato every day, and she finally ate one.”*

(id 7)

To complement quantitative and qualitative analysis, integration was found to be related to responsive finishing. A high proportion of the Chinese mothers stated that they were concerned about the amount of food and stated the use of feeding bowls to restrict portion sizes, which is a Chinese cultural feeding practice.

“I do not give them too much food to eat, keeping it under control. Any foods they eat are served in a bowl. They have their own feeding bowl, so no regardless of whether it is noodles or rice or any other foods, they eat a similar amount. Even if they’d like to eat more, I do not give them. They only eat what fits in a bowl.”

(id 16)

In this study, three children had food allergies and another three were classified as fussy eaters. Chinese mothers with the highest scores in integration showed that they were concerned about meeting nutritional requirements. Mothers stated that they did not subject their children to pressure to finish food. However, they gave a feeding bowl to their children, which was partly divergent from quantitative analysis.

“I will not push my children to eat more than they want. He usually eats up the amount of a bowl of food.”

(id 17)

#### 3.3.2. Relationship of Marginalisation with Indulgence 

During interviews, most of the Chinese mothers expressed less marginalisation in relation to indulgence coaxing and permissive feeding. In fact, the triangulation of quantitative and qualitative analysis showed that marginalisation was related to indulgence and permissive feeding. Some mothers lacked immersion in either the UK or Chinese culture. One mother was wearing outdated clothes, did not have any social media apps and was choosing foods only from advertisements aimed at children (such as on television or in newspapers). She allowed her children to eat unhealthy food if they wanted, adhered to and applied traditional Chinse medicine (TCM), and cooked complicated traditional Chinese food.

“When we go out to have dinner with our friends’ children, some children would like to eat a hamburger while others prefer Chinese food. We buy hamburgers and take it to Chinese restaurants to eat.”

(id 13)

In this study, the interview results of the Chinese mothers with lowest scores for marginalisation, showed a partial discrepancy with quantitative analysis. They showed less pampering and soothing feeding. Some mothers had not integrated into British culture and were also equally distant from the developing Chinese culture they belonged to. Despite living in England for decades, there was a lack of progress in their routine and daily lives, matched with a lack of updated feeding practices. They still obeyed some outdated Chinese CIYCFP. One mother only received information from a Chinese church. She learned from an old Chinese feeding book containing outdated advice on feeding practices. She did not demonstrate indulgence coaxing. On the contrary, she punished her child with hunger if they did not eat at regular times. (id 31) 

#### 3.3.3. Relationship of Separation with Restrictive Diet Quality

In this study, the score of separation was below the average value, which was in dissonance with interview results showed a high level of separation. The triangulation of quantitative and qualitative analysis showed that separation was related to a restrictive diet quality. The interviews showed that most of the Chinese mothers were less adjusted than their children. Some stated that since their children grew up in England and absorbed both cultures, their children did not experience dietary and culture conflict. However, these mothers displayed dietary and culture conflict, claiming to have a ‘Chinese stomach’ and finding it very hard to adjust to British dietary culture.

At an external level, this was exhibited by their strong preference for hot foods or water, while generally avoiding eating or drinking cold foods or water. Intrinsically, they kept a Chinese lifestyle in England, habitually still ate Chinese foods and maintained Chinese friendships, language, rules, beliefs, values, etc.

Although some Chinese mothers had lived in England for decades, they had not entirely adjusted to or accepted British dietary beliefs, behaviours, culture and lifestyles. Therefore, they still retained Chinese dietary culture and lifestyles, and some Chinese mothers considered Chinese dietary culture to be healthier than British culture, viewing it as ‘light’ and nutritious food.

“I control my children to eat light they grow up, I cannot continue to control this but, if I could, I would try to keep them eating light foods.”

(id 8)

*“It is important for my children to eat healthy foods, such as fruits and vegetables, which affect their health in the future. I do not allow them to choose food by themselves.”*

(id 22)

*“I do not give my children fried foods, such as fries and hamburgers. I have to cook food to take along when we are outside and rarely give them junk foods, except in the situation where we cannot cook.”*

(id 16)

*“I only buy some high-quality meat. I will not buy 3 for £10 – I choose more expensive food. I also particularly buy organic food.”*

(id 22)

#### 3.3.4. Relationship of Assimilation with CIYCFP

In this study, Chinese mothers showed less assimilation, which demonstrated agreement with the interview results. Only one Chinese mother stated, *‘I am an English woman born in China’* (id 14). Some mothers stated that their children showed assimilation to dominant foods. The majority of Chinese mothers obey western heath professionals’ suggestions for dealing with their child’s fussy eating behaviour and avoid Chinese methods. For instance,

“Fussy eating is a big problem. The health visitor taught us to constantly let children try food, not force them, and keep food in front of children. The Chinese method is to force children or mix food with sugar or other foods that children like—anything, as long as the food ends up in the children’s mouth.”

(id 27)

*“They eat western food at school. It is culturally compatible. For younger generations, it is easy to make compatible.”*

(id 4)

### 3.4. Facilitators and Barriers of Acculturation in CIYCFP

#### 3.4.1. Acculturation and Feeding Conflict

Some Chinese mothers argued that since Chinese physiology was different from British physiology, they could not adjust to British dietary practices. Instead, they favoured Chinese dietary cooking at home to take out and avoided unhealthy British food.

“We did not grow up in England. Though I gave birth to my child in England, I think his physique is Chinese. We are different to the British, especially our diet and culture.”

(id 12)

*“Though I have been living in England for decades, I still think the Chinese diet is relatively healthier. Because the physique of my children is different from the British, I must cook Chinese food to take with us when we go outside and rarely feed my children junk food.”*

(id 16)

One Chinese mother expressed feeding and culture conflict in herself as she believed that all Chinese living in England should be the same as the British. Yet, she still believed in TCM after living in Britain for more than 10 years.

……“When I gave birth, my mother said our physique is different from the British, so we should apply Chinese methods. In fact, I wonder if we are the same as those living in England.”

(id 15)

She also stated,

“I think TCM methods are useful for eczema. The British method is a temporary solution, they do not understand the mechanisms of eczema. The understanding of TCM theory on eczema might be more direct and effective than that of the British.”

(id 15)

Chinese mothers sought to assimilate and believed that those who underwent greater changes in dietary beliefs and behaviours might experience lower levels of prejudice or discrimination.

“I cooked pork bone soup, which was nutritious and very delicious. The Chinese like it very much, but people of other races argue that the smell is disgusting. It is an embarrassing cultural conflict on diet.”

(id 12)

#### 3.4.2. Balancing Western and Chinese Feeding Practices

TCM is an important part of Chinese traditional culture. While some Chinese mothers applied TCM theory in CIYCFP, large numbers of Chinese mothers balanced and integrated western medicine with TCM theory, which was another example of integration. Interviews showed that when British and Chinese information around CIYCFP caused dietary culture conflicts and contradictions, some mothers integrated them. For instance, some mothers used Chinese cooking methods with western diet and substituted Chinese soup noodles for pasta cooked in Chinese thin soup. 

One of the Chinese mothers stated she had a process for adapting British CIYCFP with her first child by applying traditional Chinese feeding practices according to advice from her mother-in-law. However, she gradually adjusted CIYCFP opinions for herself when informed of British CIYCFP advice by midwives and health visitors when she had her second and third children (id 27). Most Chinese food needs to be cooked for a longer time than British food and could be more complicated to prepare (e.g., dumplings). Because of this, some Chinese mothers opted to prepare simple, healthy and fast western foods, such as sandwiches.

Some of Chinese mothers worried about schools not preparing Chinese or Asian food and that as a result of this, they would have to adjust food preparation at home to suit the British diet.

“If we only cook Chinese food at home, my children would not eat food prepared by their school, and some Chinese foods are not allowed at school as they have said those Chinese foods are not healthy.”

(id 20)

*“I follow the school’s menu when cooking food at home. Parents have developed Chinese dietary habits and taste. Though we like Chinese food, I feed my children different kinds of food.”*

(id 17)

## 4. Discussion

The experience of acculturation is important as Chinese immigrants continue to arrive in England. How acculturation influences CIYCFP needs to be further explored. Thinking of acculturation as a force shaping CIYCFP has implications regarding equity of access and culturally appropriate health policy. To our knowledge, this is the first mixed qualitative and quantitative study to present the association between acculturation and CIYCFP among new Chinese immigrant mothers living in England, with the aim of better understanding how acculturation shapes CIYCFP. This study indicated that Chinese mothers who were better integrated were likely to respond to satiety and attention. Those inclined towards being marginalised were more likely to indulge their children, while those who were more culturally separated were more likely to restrict the food quality offered to their children. This study also indicated that Chinese immigrants balanced western and Chinese feeding practices to combat dietary and culture conflict. 

Integration is an option open to immigrants that are interested in maintaining their culture of heritage and who are involved in daily interactions with the dominant society. Immigrants can maintain cultural integrity while they seek to be a member of the heritage group but not as an integral part of the dominant society [5]. Research proposes that the best adaptation will be among immigrants who seek to integrate [6]. Integration on responsive to satiety is one of the important determinants of CIYCFP [38]. In this study, Chinese mothers with the highest scores of integration were highly responsive to satiety and attention and highly pressured their children to finish eating. WHO suggest that mothers should be responsive to the child’s clues for hunger and responsive feeding [12]. A study indicated that responsive satiety might distract infant and young child from satiety by concentrating on the accessibility of restricted foods, while excessive pressuring was believed to improve food intake in the absence of satiety [39]. Infant and young child should learn to control the amount of food and finish food based on responsiveness to internal satiety and hunger cues [40]. If children do not control their food intake, they might not regulate their own appetite. Thus, this could increase the risk of being obese later in life [41]. High integration is understood with mothers who are less authoritarian [42]; however, the children of authoritarian mothers who are able to balance demand and responsiveness tend to have lower BMIs than children with indulgent mothers [43]. 

This study showed that separated mothers had high influence and scores in restrictive diet quality. This result was consistent with a UK study which showed that Chinese immigrants had the highest restrictive feeding scores compared to other minorities [44]. Separation is when immigrants place a high value on retaining their culture of heritage while simultaneously deliberately avoiding interaction with the dominant culture [5]. Separated mothers affect their children’s development of dietary preferences and emotional eating atmospheres, which might result in encouraging children to eat [45]. Furthermore, restrictive access to particular kinds of food might improve children’s dietary preferences, while obliging children to eat certain kinds of food makes them dislike eating that food [46]. When children are restricted strongly to eat more food, they might experience an absence of hunger [47] and have fewer positive interactions with healthy eating beliefs and behaviours [9]. Controlling feeding is often measured in terms of implementing pressuring and restrictive feeding in CIYCFP [30]. Mothers with high controlling feeding are reported that their children have worse dietary preferences and higher weight [33]. In this study, high scores of pressuring and restrictive feeding were dissonant with the interview. Chinese mothers have been known to be highly controlling [48], authoritarian [49] and ingrained in traditional culture. However, this parenting style was shown to be correlated with positive health outcomes [50]. Further research is needed to emphasise whether controlling CIYCFP is beneficial or detrimental or to determine what extent controlling CIYCFP is beneficial. 

Adaptation is observed as the relatively steady shifts that happen in immigrants responding to external pressures as they gradually adopt the dominant society’s feeding practices [1]. When acculturating immigrants encounter problems, these experiences result in poor adaptation. Marginalised immigrants have few possibilities for, or interest in, cultural maintenance. This enforces cultural loss, and they also express little interest in active contact with the dominant society [5]. In this study, Chinese mothers demonstrated less marginalisation. This was correlated with low indulgence scores. These mothers might experience exclusion or discrimination [5], which might influence them to feed appropriate complementary food. This, in turn might, be a risk factor of childhood obesity [51]. To attain integration, mutual accommodation between immigrants and the dominant culture is required. This involves the acceptance of feeding beliefs and behaviours of other minorities so that all may live together as culturally distinct groups. This requires minorities to adopt the dominant society’s basic CIYCFP, while the dominant society adapts national health policy and institutions to better meet the needs of minorities. 

In this study, there was no expected association between assimilation and domains of CIYCFP. Chinese mothers had low scores in assimilation, which is defined as immigrants not maintaining the culture of heritage and instead seeking contact with the dominant society [5]. As immigrants tend to assimilate with the dominant society and achieve a greater understanding of the dominant culture, they might decrease authoritative control [52]. Research conducted in 1977 showed that Chinese immigrants were least assimilated among multiple ethnic minorities and that they did not change their lifestyles to adapt to British social expectations [53]. These new Chinese immigrants had a different socioeconomic profile from traditional sojourners, and the societies they came from had experienced rapid and significant changes in recent decades. Some immigrants experience prejudice or discrimination, thus, they are reluctant to assimilate in order to be accepted [54]. This may be a main reason for new Chinese immigrants to express less assimilation. These separated mothers indicated that their children showed assimilation. When children become assimilated, they adopt the behaviours and beliefs of the dominant culture, which may differ from their mothers’ dietary preferences. This results in difficulties in feeding for children and mothers with different food preferences [55]. With regards to separated mothers of assimilated children who may consume more daily sugary beverages and calories from fat [55], their acculturation may influence their children’s future acculturation to the dominant culture and diet.

Chinese immigrants have been reported to positively restrict the amount of unhealthy foods their children eat, considering Chinese dietary culture to be healthier than that of the British. This includes encouraging ‘light’ and nutritious food, feeding a variety of foods, maintaining a healthy and balanced diet, eating more vegetables and fruit and avoiding fried foods and sugar [56]. Other Chinese mothers did not feed their children with traditional Chinese foods supplemented by oil, salt and spices [57]. The negative acculturation theory interprets integration as follows [58]. Immigrants are more integrated in the dominant countries, so they adopt more unhealthy diets and norms that increase health risks and reduce health-protective heritage resources [59]. Immigrants’ preferences change from the heritage to dominant diet, which includes more fat, processed food and meat [60]. The high rates of obesity in the United States represent a trend that immigrants gradually reflect as they acculturate [61]. It may be interpreted that the prevalence of obesity in Chinese children is lower than other minorities [62]. Some positive beliefs and behaviours of CIYCFP were also identified in this study.

Mutual and interactive intercultural relations are reciprocal attitudes among heritage groups in a multicultural society. The process of balancing heritage and dominant culture might influence minorities to deliver complementary foods for their children. Acculturation involves resistance to and attempts to influence the dominant culture itself, which might be matched to an active or passive valence [5]. New Chinese immigrant mothers actively balance their Chinese and British culture. Acculturation in CIYCFP can be primarily internal, psychological or sociocultural in connecting the immigrants to the dominant culture, improving the reciprocity between immigrants and the dominant culture. Immigrants attempt to cope with the changes of acculturation and, thus, might achieve long-term adaptation of CIYCFP. This long-term adaptation is high variable, from poor to good. Some immigrants may not be able to remain in the dominant society, or some immigrants can adapt their diet to that of the dominant society very well. As immigrants become acculturated, their values, health beliefs and behaviours, such as food preferences, may change. Some acculturational influences on CIYCFP might increase a risk of childhood obesity among Chinese immigrants, which may change and ultimately affect their children’s health [10]. 

## 5. Strengths and Limitations

To our best knowledge, this is the first study to present preliminary findings of the association between acculturation and CIYCFP using the triangulation of quantitative and qualitative analyses. Even at this preliminary level, culturally appropriate understanding can be improved regarding multiple British racial and ethnic groups. 

The limitations of this study include the limited data regarding the internal reliability of MIRIPS. MIRIPS was validated in multiple racial and ethnic groups, including the Chinese ethnic group [5], yet low scores in internal consistency reliability can weaken the association between acculturation and CIYCFP [63]. 

A study measuring MIRIPS in people in Hong Kong found that they have higher integration scores (0.70) compared with those we found for our sample from mainland China (0.448). The Chinese are a diverse group with huge differences in political and economic factors, culture and beliefs. Compared to those from mainland China, the cultures of Chinese immigrants from Hong Kong, Malaysia, etc. are more in line with those of western societies, and they may therefore experience less acculturation issues. 

The sample of this study was not nationally representative. Though authors attempted to access an extensive Chinese group, participants were recruited through snowball sampling, which limited the generalisability to all new Chinese immigrant mothers living in other parts of England. This limits the conclusions that can be made about the directionality of the relationship between acculturation and CIYCFP. 

Though a researcher with proficiency in Mandarin and English was involved, language limitations might have resulted in selection bias. Further research is needed to explore the association between acculturation and CIYCFP within a larger sample size and to corroborate findings with other minorities in England.

## 6. Conclusions

This study presented the association between acculturation and CIYCFP among new Chinese immigrant mothers living in England. This study indicated that Chinese mothers who were better integrated were more likely to respond to satiety and attention. Those inclined to be marginalised were more likely to indulge their children, while those who were more culturally separated were more likely to restrict the quality of foods offered to their children. We also determined facilitators and barriers to CIYCFP that were influenced by acculturation. Balancing the culture of heritage and the dominant society might influence new Chinese immigrant mothers to deliver complementary foods. Further studies are required to construct culturally appropriate interventions to tailor IYCF to minorities. This study also indicates that Chinese immigrants balanced western and Chinese feeding practices to combat feeding and culture conflict. This study presented preliminary findings regarding the association between acculturation and CIYCFP, which can improve cultural appropriateness for minorities. Interventions on CIYCFP should involve communication and decision-making with Chinese mothers to integrate strategies that promote optimal feeding practices. The findings can be applied to implementing community-based interventions on CIYCFP and conducting further studies to explore intervention programs to tailor CIYCFP with consideration for acculturation in minorities.

## Figures and Tables

**Table 1:** IFSQ in laissez-faire, pressuring, and restrictive scores (*n* = 32).

	The IFSQ in Laissez-Faire, Pressuring, and Restrictive Scores	Mean	SD
	**Laissez-Faire**	2.68	0.73
	**Attention**	2.43	0.96
***behaviour***	*When (name of child) has/had a bottle, I prop/propped it up*	*2.29*	*1.14*
	*(Child) watches TV while eating*		
	*I watch TV while feeding (child)*		
***belief***	*I think it is okay to prop an infant’s bottle*	*2.63*	*1.18*
	*It’s okay for a toddler to walk around while eating as long as s/he eats*		
	**Diet quality**	2.68	0.73
***behaviour***	*I keep track of what food (child) eats*	*2.93*	*0.93*
	*I keep track of how much food (child) eats*		
	*I make sure (child) does not eat sugary food like candy, ice cream, cakes or cookies*		
	*I make sure (child) does not eat junk food like potato chips, Doritos and cheese puffs*		
***belief***	*A toddler should be able to eat whatever s/he wants for snacks*	*2.17*	*1.13*
	*A toddler should be able to eat whatever s/he wants when eating out at a restaurant*		
	**Pressuring**	2.81	0.50
	**Finishing**	3.16	0.51
***behaviour***	*Try to get (child) to finish his/her food*	*3.03*	*0.46*
	*If (child) seems full, encourage to finish anyway*		
	*Try to get (child) to finish breastmilk or formula*		
	*Try to get (child) to eat even if not hungry*		
	*Insist retry new food refused at same meal*		
	*Praise after each bite to encourage finish food*		
***belief***	*Important for toddler finish all food on his/her plate*	*3.55*	*0.96*
	*Important for infant finish all milk in his/her bottle*		
	**Cereal**	2.85	0.81
***behaviour***	*Give/gave (child) cereal in the bottle*	*2.78*	*1.29*
***belief***	*Cereal in bottle helps infant sleep through the night*	*2.87*	*0.88*
	*Putting cereal in bottle good to help infant feel full*		
	*An infant <6 months needs more than formula or breastmilk to be full*		
	*An infant <6 months needs more than formula or breastmilk to sleep through the night*		
	**Soothing**	2.05	0.90
***behaviour***	*When (child) cries, immediately feed him/her*	*2.19*	*1.06*
***belief***	*Best way to make infant stop crying is to feed*	*2.01*	*0.98*
	*Best way to make toddler stop crying is to feed*		
	*When infant cries, usually means s/he needs to be fed*		
	**Restrictive**	3.12	0.64
	**Amount**	3.41	0.94
***behaviour***	*I carefully control how much (child) eats*	*3.28*	*0.99*
	*I am very careful not to feed (child) too much*		
***belief***	*Important parent has rules re: how much toddler eats*	*3.55*	*1.15*
	*Important parent decides how much infant should eat*		
	**Diet quality**	2.95	0.81
***behaviour***	*I let (child) eat fast food*	*2.16*	*1.02*
	*I let (child) eat junk food*		
***belief***	*A toddler should never eat fast food*	*3.26*	*1.04*
	*An infant should never eat fast food*		
	*A toddler should never eat sugary food like cookies*		
	*A toddler should never eat junk food like chips*		
	*A toddler should only eat healthy food*		

SD (Standard Deviation).

**Table 2:** IFSQ in responsive and indulgence scores (*n* = 32).

	The IFSQ in Responsive and Indulgence Scores	Mean	SD
	**Responsive**	3.96	0.72
	**Satiety**	4.00	0.80
***Behaviour***	*(Child) lets me know when s/he is full*	*4.06*	*0.71*
	*(Child) lets me knows when s/he is hungry*		
	*I let (child) decide how much to eat*		
	*I pay attention when (child) seems to be telling me that s/he is full or hungry*		
	*I allow (child) to eat when s/he is hungry*		
***Belief***	*Child knows when s/he is full*	*3.84*	*1.18*
	*Child knows when hungry, needs to eat*		
	**Attention**	3.90	0.88
***Behaviour***	*Talk to (child) to encourage to drink formula/breastmilk*	*3.80*	*0.94*
	*Talk to (child) to encourage him/her to eat*		
	*Show (child) how to eat by taking a bite or pretending to*		
	*I will retry new foods if they are rejected at first*		
***Belief***	*Important to help or encourage a toddler to eat*	*4.28*	*0.92*
	**Indulgence**	2.15	0.89
	**Permissive**	*2.* *20*	0.93
***Behaviour***	*Allow child watch TV while eating if s/he wants*	*2.23*	*0.94*
	*Allow child to eat fast food if s/he wants*		
	*Allow child to drink sugared drinks/soda if s/he wants*		
	*Allow child to eat desserts/sweets if s/he wants*		
***Belief***	*Toddlers should be allowed to watch TV while eating if they want*	*2.18*	*1.07*
	*Toddlers should be allowed to eat fast food if they want*		
	*Toddlers should be allowed to drink sugared drinks/soda if they want*		
	*Toddlers should be allowed to eat desserts/sweets if they want*		
	**Coaxing**	2.18	1.00
***Behaviour***	*Allow child watch TV while eating to make sure s/he gets enough*	*2.30*	*1.01*
	*Allow child to eat fast food to make sure s/he gets enough*		
	*Allow child to drink sugared drinks/soda to make sure s/he gets enough*		
	*Allow child to eat desserts/sweets to make sure s/he gets enough*		
***Belief***	*Toddlers should be allowed to watch TV while eating to make sure they get enough*	*2.06*	*1.14*
	*Toddlers should be allowed to eat fast food to make sure they get enough*		
	*Toddlers should be allowed to drink sugared drinks/soda to make sure they get enough*		
	*Toddlers should be allowed to eat desserts/sweets to make sure they get enough*		
	**Soothing**	2.00	1.00
***Behaviour***	*Allow child watch TV while eating to keep him/her from crying*	*2.02*	*1.12*
	*Allow child to eat fast food to keep him/her from crying*		
	*Allow child to drink sugared drinks/soda to keep him/her from crying*		
	*Allow child to eat desserts/sweets to keep him/her from crying*		
***Belief***	*Toddlers should be allowed to watch TV while eating to keep them from crying*	*1.98*	*0.99*
	*Toddlers should be allowed to eat fast food to keep them from crying*		
	*Toddlers should be allowed to drink sugared drinks/soda to keep them from crying*		
	*Toddlers should be allowed to eat desserts/sweets to keep them from crying*		
	**Pampering**	2.21	1.06
***Behaviour***	*Allow child watch TV while eating to keep him/her happy*	*2.57*	*0.55*
	*Allow child to eat fast food to keep him/her happy*		
	*Allow child to drink sugared drinks/soda to keep him/her happy*		
	*Allow child to eat desserts/sweets to keep him/her happy*		
***Belief***	*Toddlers should be allowed to watch TV while eating to keep them happy*	*1.97*	*0.92*
	*Toddlers should be allowed to eat fast food to keep them happy*		
	*Toddlers should be allowed to drink sugared drinks/soda to keep them happy*		
	*Toddlers should be allowed to eat desserts/sweets to keep them happy*		

SD (Standard Deviation).

**Table 3:** The association of acculturation with five domains of CIYCFP (*n* = 32).

	Mean (SD)	Laissez-Faire	Pressuring	Restrictive	Responsive	Indulgence
Ethnic identity	4.36(0.70)	−0.399 *	0.286	0.306	0.258	−0.114
National identity	2.56(1.01)	0.375 *	0.091	−0.380 *	−0.047	0.090
Integration	4.44(0.62)	−0.022	0.175	0.068	0.339	−0.111
Assimilation	1.79(0.57)	0.131	0.153	−0.140	0.014	0.008
Separation	2.03(0.88)	−0.171	0.144	0.307	0.091	0.249
Marginalisation	1.67(0.73)	0.088	0.250	0.076	−0.018	0.392 *
Multicultural ideology	3.68(0.42)	−0.032	−0.160	−0.182	0.235	−0.504 **
Ethnic tolerance	3.44(0.47)	0.018	−0.500 **	−0.128	0.193	−0.275
Attitude on social equality	3.56(0.62)	−0.011	−0.215	−0.061	−0.366 *	−0.233

* Correlation is significant at the 0.05 level (2-tailed), ** Correlation is significant at the 0.01 level (2-tailed). CIYCFP (Complementary Infant and Young child Feeding Practices); SD (Standard Deviation).

**Table 4:** The association of acculturation with subscales of five domains of CIYCFP (*n* = 32).

	LF At	LF DQ	PR F	PR Ce	PR So	RS Am	RS DQ	RP Sa	RP At	ID Pe	ID Co	ID So	ID Pa
Ethnic identity	−0.016	−0.399 *	0.194	0.128	0.309	0.397 *	0.116	0.097	0.381 *	−0.040	−0.161	−0.085	−0.114
National identity	0.054	0.375 *	0.172	0.101	−0.097	−0.264	−0.295	−0.002	−0.089	0.056	−0.065	0.203	0.120
Integration	−0.038	−0.022	0.280	0.168	−0.095	0.161	−0.023	0.368 *	0.196	−0.033	−0.239	−0.050	−0.071
Assimilation	−0.064	0.131	0.051	0.227	0.047	0.063	−0.216	0.155	−0.168	−0.014	0.026	−0.021	0.036
Separation	0.258	−0.171	0.120	−0.057	0.264	0.037	0.355*	0.182	−0.052	0.200	0.302	0.155	0.228
Marginalisation	0.317	0.088	0.228	0.042	0.280	−0.025	0.110	0.101	−0.163	0.312	0.432 *	0.317	0.330
Multicultural ideology	−0.173	−0.032	−0.058	−0.142	−0.151	−0.104	−0.156	0.358 *	0.006	−0.520 **	−0.567 **	−0.276	−0.436 *
Ethnic tolerance	−0.143	0.018	−0.551 **	−0.353 *	−0.150	0.018	−0.171	0.076	0.280	−0.251	−0.397 *	−0.243	−0.098
Attitude on social equality	−0.191	−0.011	−0.162	−0.142	−0.162	−0.085	−0.019	0.196	0.466 **	−0.132	−0.457 **	−0.154	−0.091

* Correlation is significant at the 0.05 level (2-tailed), ** Correlation is significant at the 0.01 level (2-tailed). CIYCFP- Complementary Infant and Young child Feeding Practices; LF-Laissez-Faire; At-Attention; DQ-Diet Quality; PR-Pressuring; F-Finishing; Ce-Cereal; So-Soothing; RS-Restrictive; Am-Amount; RP-Responsive; Sa-Satiety; ID-Indulgence; Pe-Permissive; Co-Coaxing; Pa-Pampering.

**Table 5:** The association of acculturation with behaviours of domains of CIYCFP (*n* = 32).

	LF At	LF DQ	PR F	PR Ce	PR So	RS Am	RS DQ	RP Sa	RP At	ID Pe	ID Co	ID So	ID Pa
Ethnic identity	0.029	−0.461 **	0.155	0.064	0.358 *	0.340	−0.087	0.189	0.337	0.036	−0.040	0.043	−0.004
National identity	−0.088	0.466**	0.246	0.247	0.109	−0.277	−0.073	0.105	−0.110	0.076	−0.077	0.225	0.060
Integration	−0.038	−0.107	0.167	−0.038	0.142	0.135	−0.132	0.440*	0.142	-0.023	−0.224	−0.044	−0.032
Assimilation	−0.010	0.215	0.042	0.232	0.161	0.037	−0.254	0.157	−0.159	−0.141	−0.150	−0.121	0.024
Separation	0.256	−0.325	0.131	−0.065	0.192	−0.006	0.089	0.131	−0.039	0.076	0.274	0.034	0.290
Marginalisation	0.325	−0.032	0.244	0.050	0.240	−0.114	0.072	0.085	−0.168	0.147	0.353 *	0.195	0.418 *
Multicultural ideology	−0.181	0.033	−0.091	0.127	0.074	−0.046	−0.273	0.427 *	−0.060	−0.403 *	−0.481 **	−0.187	−0.469 **
Ethnic tolerance	−0.123	0.024	−0.482 **	−0.219	−0.192	0.068	−0.204	0.083	0.267	−0.231	−0.406 *	−0.170	−0.317
Attitude on social equality	−0.281	0.034	−0.135	−0.083	−0.126	−0.019	−0.209	0.317	−0.452 **	−0.045	−0.421 *	−0.072	−0.228

* Correlation is significant at the 0.05 level (2-tailed), ** Correlation is significant at the 0.01 level (2-tailed). CIYCFP- Complementary Infant and Young child Feeding Practices; LF-Laissez-Faire; At-Attention; DQ-Diet Quality; PR-Pressuring; F-Finishing; Ce-Cereal; So-Soothing; RS-Restrictive; Am-Amount; RP-Responsive; Sa-Satiety; ID-Indulgence; Pe-Permissive; Co-Coaxing; Pa-Pampering.

**Table 6:** The association of acculturation with beliefs of domains of CIYCFP (*n* = 32).

	LF At	LF DQ	PR F	PR Ce	PR So	RS Am	RS DQ	RP Sa	RP At	ID Pe	ID Co	ID So	ID Pa
Ethnic identity	−0.075	−0.019	0.192	0.123	0.250	0.359 *	0.160	−0.055	0.450 **	−0.101	−0.245	−0.222	−0.113
National identity	0.238	−0.036	0.016	0.026	−0.158	−0.196	−0.293	−0.163	0.021	0.030	−0.046	0.157	0.195
Integration	−0.022	0.132	0.359 *	0.206	−0.168	0.147	0.026	0.208	0.358 *	−0.037	−0.218	−0.051	−0.025
Assimilation	−0.116	−0.099	0.048	0.175	−0.001	0.071	−0.135	0.130	−0.160	0.100	0.179	0.094	−0.052
Separation	0.152	0.202	0.070	−0.041	0.255	0.067	0.352*	0.234	−0.091	0.280	0.283	0.276	0.101
Marginalisation	0.172	0.224	0.138	0.030	0.258	0.058	0.092	0.112	−0.099	0.412 *	0.439 *	0.422 *	0.224
Multicultural ideology	−0.088	−0.117	0.007	−0.208	−0.212	−0.131	−0.063	0.203	0.272	−0.547 **	−0.561 **	−0.348	−0.348
Ethnic tolerance	−0.112	−0.004	−0.488 **	−0.324	−0.115	−0.029	−0.106	0.055	0.253	−0.233	−0.331	−0.300	−0.136
Attitude on social equality	0.020	−0.078	−0.153	−0.132	−0.154	−0.124	0.061	−0.013	0.389 *	−0.190	−0.422*	−0.231	−0.087

** Correlation is significant at the 0.01 level (2-tailed), * Correlation is significant at the 0.05 level (2-tailed). CIYCFP- Complementary Infant and Young child Feeding Practices; LF-Laissez-Faire; At-Attention; DQ-Diet Quality; PR-Pressuring; F-Finishing; Ce-Cereal; So-Soothing; RS-Restrictive; Am-Amount; RP-Responsive; Sa-Satiety; ID-Indulgence; Pe-Permissive; Co-Coaxing; Pa-Pampering.
